# The Trouser Technique: A Novel Approach for Peri-Implant Soft Tissue Augmentation

**DOI:** 10.3390/jcm14144974

**Published:** 2025-07-14

**Authors:** Pablo Pavón, Carla Fons-Badal, Natalia Pérez-Rostoll, Jorge Alonso-Pérez-Barquero, María Fernanda Solá-Ruiz, Rubén Agustín-Panadero

**Affiliations:** 1 Private Practice, 46005 Seville, Spain; pavon@pablopavon.com; 2 Department of Oral Medicine, Faculty of Medicine and Dentistry, University of Valencia, C/Gascó Oliag, 1, 46010 Valencia, Spain; nataliarostoll99@icloud.com (N.P.-R.); jorgealonso86@gmail.com (J.A.-P.-B.); ruben.agustin@uv.es (R.A.-P.)

**Keywords:** connective tissue graft, peri-implant mucosa, tunneling, dental implants

## Abstract

**Background/Objectives**: Peri-implant mucosa plays a key role in both peri-implant health and aesthetics. Differences in contour and color between implants and natural teeth can negatively affect patient satisfaction, while soft tissue deficiency may lead to complications such as peri-implantitis. Peri-implant plastic surgery aims to improve these conditions. The objective of this study is to describe the trouser-shaped connective tissue graft technique designed to enhance vestibular and interproximal peri-implant tissue volume in a single surgical procedure, and to assess its effectiveness and morbidity. **Methods**: Ten patients requiring soft tissue augmentation in edentulous areas prior to delayed implant placement were selected. Intraoral scanning was performed before and 6 months after treatment to evaluate tissue thickness gain. **Results**: Significant soft tissue volume gain was observed at both the coronal (mean: 2.74 mm with a 95% confidence interval of 2.21–3.26 mm) and vestibular (mean: 2.79 mm with a 95% confidence interval of 2.24–3.35 mm) levels in all analyzed positions (*p* < 0.001). The procedure exhibited low morbidity, with minimal complications and discomfort reported by the patients. **Conclusions**: The trouser-shaped connective tissue graft technique is effective in increasing peri-implant soft tissue. It allows for vestibular and interproximal tissue augmentation in a single procedure, minimizing tissue contraction and morbidity. This technique could be a predictable and minimally invasive alternative for managing volume deficiencies in peri-implant tissues, particularly in aesthetic areas.

## 1. Clinical Relevance

The trouser-shaped connective tissue graft technique is a predictable, minimally invasive procedure that enables the augmentation of both vestibular and interproximal peri-implant tissues in a single intervention. This reduces postoperative morbidity and promotes greater patient acceptance. The technique is especially beneficial in the anterior zone, where soft tissue volume is critical to clinical success.

## 2. Introduction

The peri-implant mucosa plays a fundamental role in aesthetics and in maintaining health around implants. To prevent inflammation and achieve satisfactory aesthetic results, an adequate band of keratinized mucosa around the implant is essential [1,2]. Regarding aesthetics, differences in contour and color between the implant and natural teeth can significantly affect patient satisfaction [3]. Increased soft tissue thickness improves contour, color, and provides dimensional stability [4,5]. Furthermore, after tooth loss, the interproximal tissue level is critical, and changes in interproximal architecture and papilla recession often occur [6,7].

In terms of health maintenance, the peri-implant mucosa has a barrier function that helps prevent bacterial invasion. Certain studies indicate the need for a minimum of 2 mm of keratinized tissue to prevent inflammatory and infectious processes such as mucositis or peri-implantitis [8]. When its thickness is insufficient, bacterial biofilm accumulates more easily, increasing the probability of infection [3,9]. Numerous studies have shown that implants with insufficient keratinized tissue exhibit higher rates of peri-implantitis, greater biofilm accumulation, soft tissue inflammation, recession, patient discomfort, and require more frequent periodontal maintenance [10,11,12,13]. Additionally, the vertical volume of peri-implant soft tissues has been linked to the prevention of bone remodeling [13,14].

Techniques to enhance soft tissue conditions around both teeth and implants have been documented over many years, notably by Edel in 1974, who compared different grafting approaches. A statistically significant increase in the width of keratinized attached gingiva was observed at 6 months [15]. Travelli et al. in 2021 conducted a review comparing different types of grafts, concluding that bilaminar grafts with type I collagen or acellular dermal matrix showed greater soft tissue gain, while the partial thickness flap technique combined with a connective tissue graft proved more effective in increasing the width of keratinized mucosa (KM) [2]. A subsequent review by Stefanini et al. in 2023 indicated that locations where soft tissue augmentation was performed had greater volumetric stability over time for both peri-implant mucosa and marginal bone, whereas implants where augmentation was not performed sometimes experienced apical displacement of the gingival margin [16]. Therefore, peri-implant plastic surgery has improved both the aesthetic appearance and the quality of life of patients [17].

If we focus on the papilla, a large number of techniques exist for its reconstruction, such as its repositioning with regeneration procedures [18] or through tunneling procedures [19]. However, papilla deficiency around implants is relatively common, and the treatment of these aesthetic complications is considered a challenge in the literature due to its limited predictability [20,21,22]. Occasionally, multiple interventions are necessary to obtain satisfactory results both buccally and at the papilla level.

The objective of this article is to describe the Trouser Technique, a connective tissue graft technique that aims to improve the volume of peri-implant tissues buccally and interproximally with a single surgical intervention.

## 3. Materials and Methods

Ten patients requiring soft tissue augmentation in the edentulous area where a dental implant was to be placed in a delayed manner were selected. The treatment and data analysis were performed at the Department of Stomatology, University of Valencia. Inclusion criteria included adult patients requiring the placement of a single implant in the anterior sector in an already healed edentulous area with adjacent, periodontally healthy teeth, with sufficient bone to place the implant (measured by CBCT) but needing soft tissue volume augmentation both buccally and interproximally. Exclusion criteria were smoking patients; those requiring bone regeneration for implant placement; those with health problems or medication contraindicating the intervention or taking drugs that could affect gingival anatomy, such as phenytoin, cyclosporines, or nifedipine; or inability to sign informed consent. The study protocol, which fulfilled guidelines established in the Declaration of Helsinki for experiments involving human subjects, was approved by the University of Valencia Ethics Committee for Research Conducted on Humans (2024-ODON-3712410). All participants were provided with full information about the study protocol, and all provided their informed consent to take part.

### 3.1. Clinical Records and Treatment

**Figure 1:**
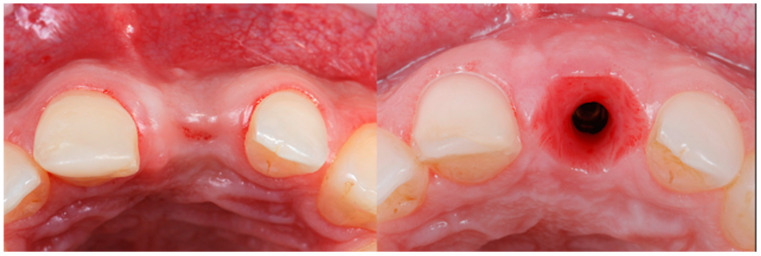
Occlusal view pre- and 6 months post-graft that shows soft tissue volume gain.

**Figure 2:**
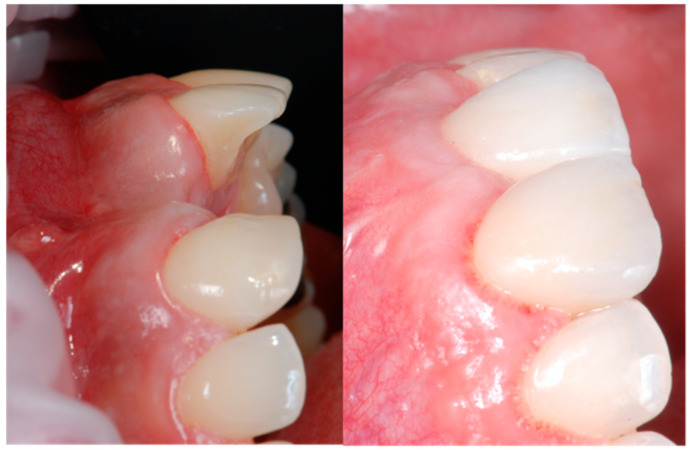
Profile with pre- and 6 months post-graft that shows soft tissue volume gain.

**Figure 3:**
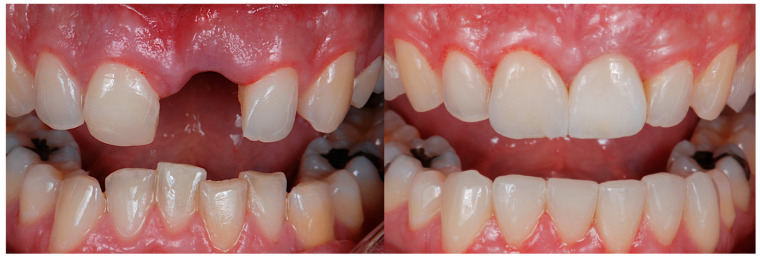
Pre- and 6 months post-graft frontal image. Gingival inflammation was noted on the tooth adjacent to the graft, presumably related to the newly placed veneer.

All the selected patients had keratinized tissue in the edentulous area, but the profile at the vestibular level was sunken (Figure 1), which compromised the aesthetics and adequate brushing of the future restoration. A pre-surgical scan of the area was performed using the Medit^®^ i700 scanner (version 3.3.3, 2024, Seoul, Republic of Korea) to obtain an STL file of the patient’s initial condition. A delayed implant placement was then carried out using the MIS^®^ C1 implant system (MIS Implants Technologies Ltd., Bar-Lev Industrial Park, Karmiel, Israel) with the MGUIDE (MIS^®^) guided surgery system, using a flapless approach. The implant was placed subcrestally between 0.5 and 1 mm, and the trouser-shaped connective tissue graft technique was simultaneously applied with a de-epithelialized connective tissue graft obtained from the tuberosity. Bone grafting was not performed as there was sufficient autologous bone for implant placement (measured by CBCT). After 6 months, a new scan was performed to assess soft tissue gain, considering whether the tissues had fully matured. Pre- and post-operative photographs were also taken to document the evolution of each case (Figure 2 and Figure 3).

Morbidity was assessed by clinical evaluation and a patient questionnaire about discomfort and limitations in their quality of life during the months following grafting. A 5-point Likert scale was used, where 0 was no discomfort and 5 was severe discomfort. Eight questions were asked regarding postoperative pain, medication, swelling, bleeding, functional limitations (eating, speaking, and brushing), and general perception.

### 3.2. Graft Technique Description

The trouser-shaped connective tissue graft technique is a mucogingival surgical procedure designed to increase soft tissue volume both in the vestibular area and papillae during implant placement surgery via tissue tunneling.

**Figure 4:**
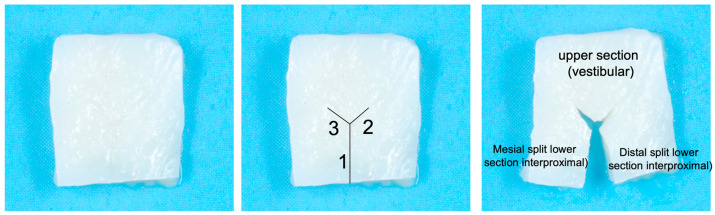
The first image is the de-epithelialized tuberosity graft, the second shows the incisions that will be made (1: central incision, 2 and 3: small 45° incisions), and the third shows the incisions already made and the parts into which the graft is divided.

**Figure 5:**
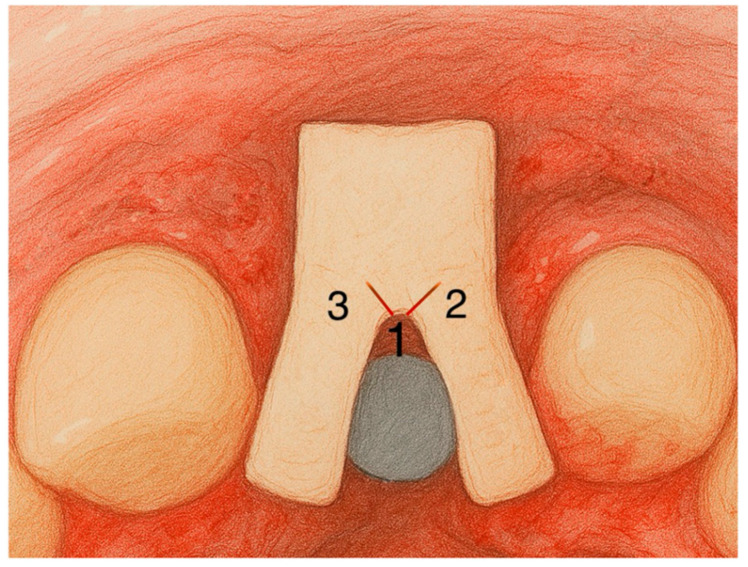
Graft positioning diagram illustrating the position of the graft of the graft in the recipient site. Number s 1, 2 and 3 are the incisions.

**Figure 6:**
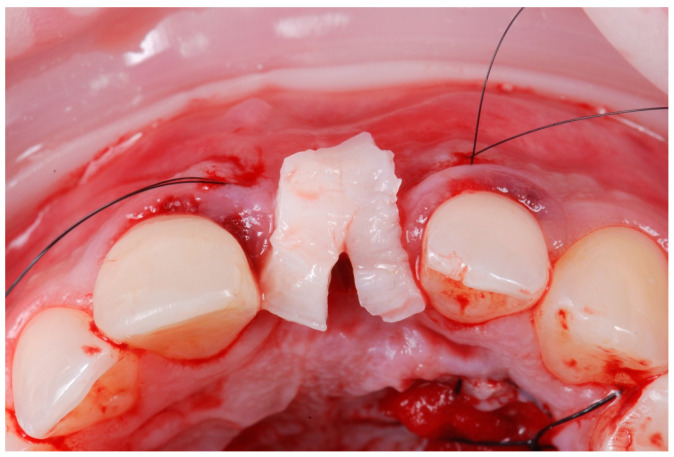
Graft from tuberosity positioned before insertion to check the position.

Harvesting and preparation of the graft: The preferred donor site is the tuberosity due to its thickness and tissue density. If this area is not suitable, a de-epithelialized free graft from the palate may be used. A rectangular graft is harvested with a thickness ranging from 1.5 to 3 mm, and three incisions are made (Figure 4): (1) a central incision dividing part of the graft into two and (2) two small 45° incisions to increase mobility of the split section. The dimensions of the graft and incisions vary depending on the treatment site. The graft consists of an upper (vestibular) and a split lower section. The upper section is positioned vestibularly, while each split limb of the graft is tunneled interproximally beneath each papilla, extending palatally (Figure 5 and Figure 6). The design depends on donor tissue availability and the edentulous space. The length of each limb is determined by the vestibulo–palatal width of the edentulous area (Video S2).

Recipient site preparation involves (1) partial-thickness tunneling of the vestibular area extending mesio–distally to the adjacent teeth and down to the vestibular depth, cutting muscle fibers to release tension; (2) intrasulcular incision on adjacent teeth and papillae tunneling by introducing the scalpel through the implant hole at 1.5–2 mm depth until it makes contact with the adjacent tooth, connecting the vestibular area with the interproximal areas; and (3) palatal tunneling from the mesial to the distal aspect of the edentulous area to mobilize the papillae and optimally position the limbs. This is performed using an ophthalmic scalpel (Equipsa^®^, Madrid, Spain) and tunnel elevators (Schwert^®^, Tuttlingen, Germany).

**Figure 7:**
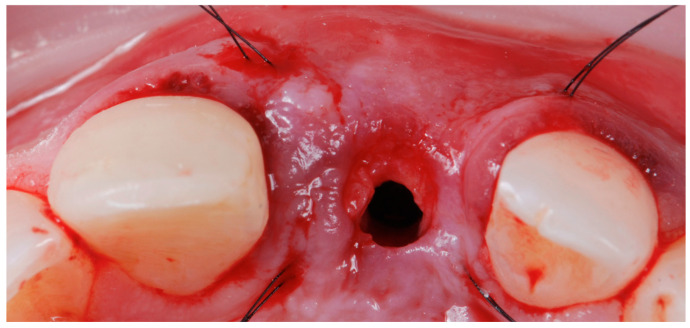
Graft inserted with the palatine sutures untied.

Graft placement and fixation: The split section is first inserted under the tunneled flap and fixed using non-resorbable 6/0 polyvinylidene fluoride sutures (Seralene^®^, Osteogenos, Madrid, Spain) at the mesio–palatal and disto–palatal aspects. Sutures are not tied until the healing abutment or provisional crown is placed, to allow for adjustment. Next, the vestibular section is positioned (Figure 7). Finally, the abutment or provisional crown is installed, the graft is adjusted by pulling the palatal sutures to optimize volume gain, and the sutures are tied (Video S1).

Healing abutment or provisional crown placement: After screwing in the provisional or healing abutment, it is unscrewed to verify proper graft seating. If displaced, the graft is repositioned using a probe. The emergence profile of the provisional should be as concave and narrow as possible to accommodate the graft during healing and maturation.

**Figure 8:**
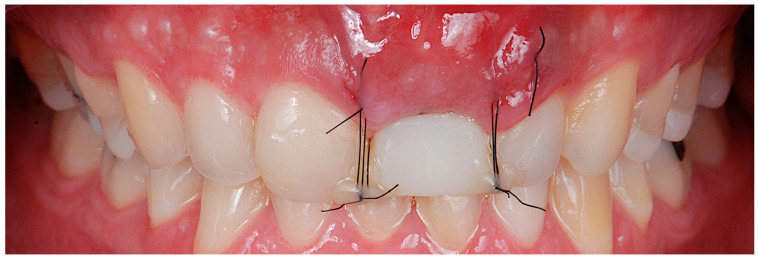
Frontal image of the graft with the 6/0 coronal traction suture and the temporary crown.

A 6/0 coronal traction suture is then placed to gently stabilize the graft, avoiding excessive traction and ischemia-related necrosis (Figure 8).

### 3.3. Digital Protocol

STL files were analyzed using Geomagic Wrap 2021 (Geomagic Verify medTM, Geomagic, Morrisville, NC, USA) and GOM Inspect 2018 (GOM GmbH, Braunschweig, Germany).

Initial STL files (STL1) were aligned with post-surgical models (STL2) using a best-fit algorithm in Geomagic Wrap 2021. Only unaltered regions, specifically, the teeth, were used for alignment to ensure greater stability and precision, while the gingival areas were excluded. Each aligned object was then exported in binary STL format and imported into GOM Inspect 2018 for measurements.

**Figure 9:**
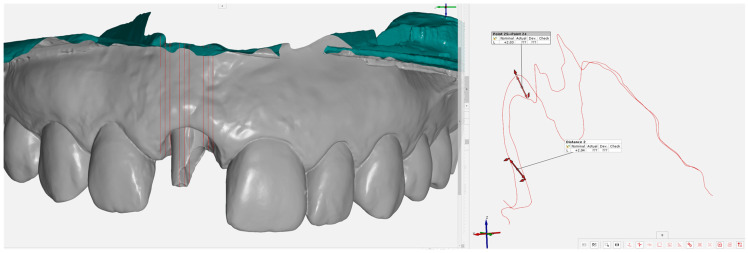
Perpendicular sections and measurement of soft tissue increment.

To measure volumetric changes in the gingiva, a reference plane corresponding to the y-axis was used to generate a longitudinal section through the center of the edentulous area in the bucco–palatal direction. Parallel sections were then created every 0.5 mm along the entire edentulous area. The study area was divided into three zones: mesial papilla, the central zone, and distal papilla, each further subdivided into mesial, medial, and distal sections for a total of nine sections (Figure 9). In each section, two measurements were taken, one at the coronal level and another at the vestibular level, by measuring the distance between corresponding points on STL1 and STL2.

**Figure 10:**
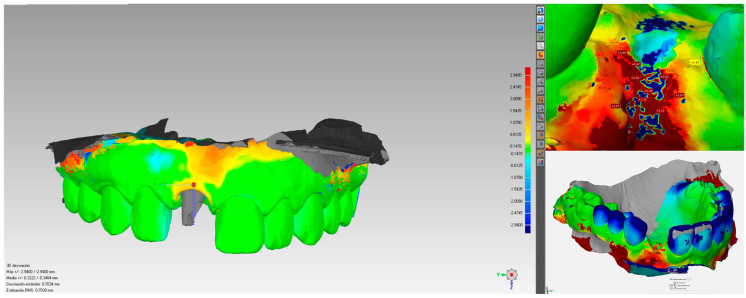
Color heat map with volumetric change.

Additionally, the software allowed for the generation of a color heat map to visually compare volumetric differences between initial and final models, clearly highlighting areas with soft tissue volume gain (Figure 10).

### 3.4. Statistical Analysis

A descriptive analysis of tissue gain was performed, and normality was assessed using the Kolmogorov–Smirnov test. To analyze the effect of location and zone on the dependent variable, a generalized estimating equation (GEE) linear model was applied. Beta regression coefficients with 95% confidence intervals (CI) were obtained. The significance level was set at α = 0.05, with a statistical power of 66.4%. The statistical software utilized for data analysis was SPSS Statistics, version 30.0.0, developed by IBM Corp. in Armonk, NY, USA.

## 4. Results

The study sample included ten patients. A total of 90 sections of observations (10 patients × 3 locations × 3 zones) were analyzed across three anatomical locations (mesial papilla, central zone, and distal papilla), each subdivided into mesial, medial, and distal segments.

A statistically significant gain in soft tissue volume 6 months post-graft was observed at both the coronal and vestibular levels in all positions analyzed (*p* < 0.001). At the coronal level, the overall average gain was 2.74 mm (95% CI: 2.21–3.26 mm, *p* < 0.001). When stratified by location, the mesial papilla demonstrated an average gain of 2.66 mm (95% CI: 2.23–3.10 mm, *p* < 0.001), the medial zone demonstrated 2.79 mm (95% CI: 2.24–3.33 mm, *p* < 0.001), and the distal papilla demonstrated 2.76 mm (95% CI: 2.16–3.37 mm, *p* < 0.001). An analysis by zone revealed average gains of 2.63 mm (95% CI: 2.12–3.13 mm, *p* < 0.001) for the mesial zone, 2.83 mm (95% CI: 2.33–3.32 mm, *p* < 0.001) for the medial zone, and 2.76 mm (95% CI: 2.19–3.33 mm, *p* < 0.001) for the distal zone (Table 1).

Similarly, at the buccal level, the overall average gain was 2.79 mm (95% CI: 2.24–3.35 mm, *p* < 0.001). By location, the mesial papilla exhibited an average gain of 2.74 mm (95% CI: 2.29–3.19 mm, *p* < 0.001), the medial zone exhibited 2.88 mm (95% CI: 2.36–3.39 mm, *p* < 0.001), and the distal papilla exhibited 2.77 mm (95% CI: 2.07–3.46 mm, *p* < 0.001). Zone-specific averages were 2.71 mm (95% CI: 2.17–3.25 mm, *p* < 0.001) for the mesial zone, 2.87 mm (95% CI: 2.38–3.36 mm, *p* < 0.001) for the medial zone, and 2.81 mm (95% CI: 2.19–3.42 mm, *p* < 0.001) for the distal zone (Table 1).

**Figure 11:**
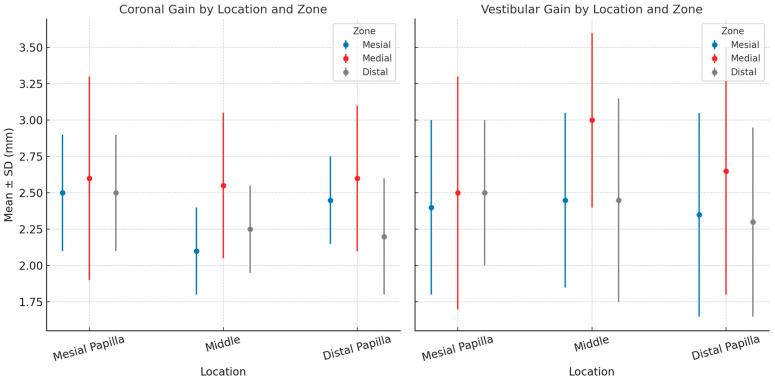
Graph illustrating tissue gain in coronal and vestibular areas.

The following figure (Figure 11) represents mean ± SD of coronal (left) and vestibular (right) gain.

**Figure 12:**
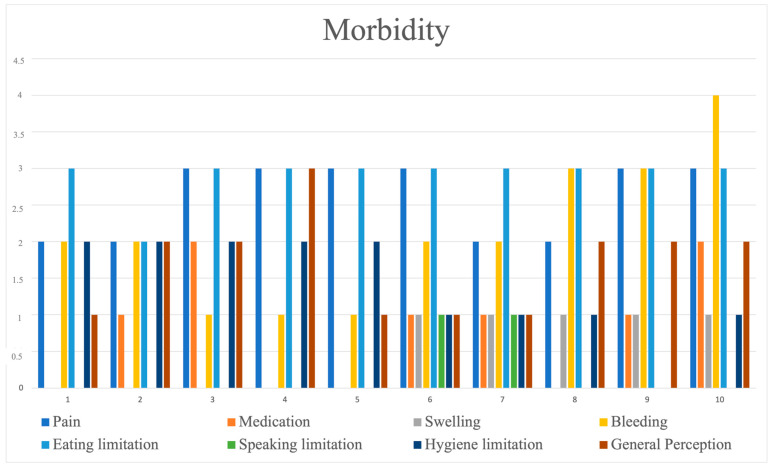
Distribution of patient responses to the eight postoperative morbidity questionnaire items, which were assessed using a 5-point Likert scale ranging from 0 (none) to 5 (very severe).

An analysis of the postoperative morbidity questionnaire revealed that although functional limitations in mastication and some pain were common experiences, the overall morbidity perceived by patients was manageable and generally of low intensity (Figure 12). The total morbidity composite score (summing up responses to the eight questions, with a maximum of 40 points) had a mean of 12.7 (standard deviation [SD] = 2.05), with a range of 10 to 16.

## 5. Discussion

The use of soft tissue grafts around implants to improve aesthetics and peri-implant stability is widely supported by the literature [23,24]. Authors such as Stefanini et al. in 2016 and Wang et al. in 2018 demonstrate the efficacy of submarginal connective tissue grafts for these purposes [25,26].

**Figure 13:**
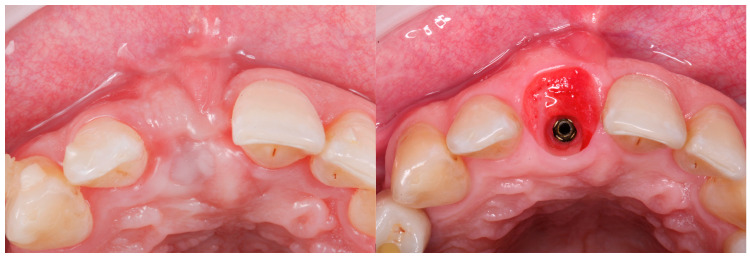
Occlusal view pre- and 6 months post-graft.

**Figure 14:**
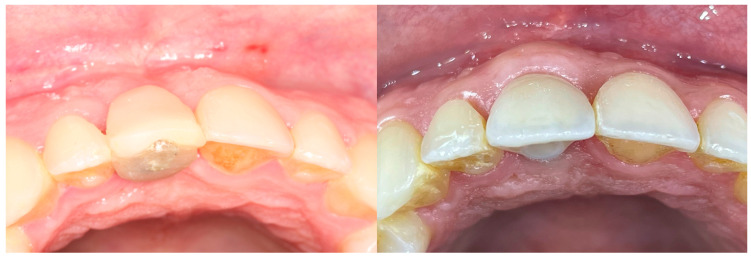
Occlusal view pre- and 6 months post-graft.

**Figure 15:**
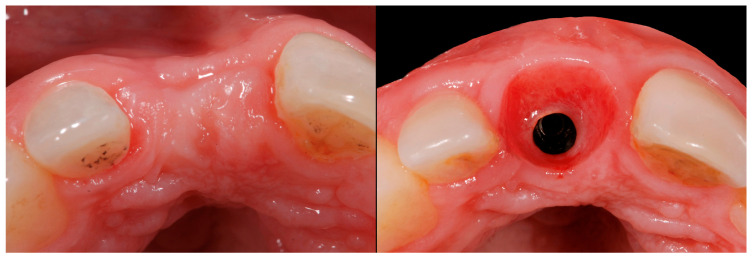
Occlusal view pre- and 6 months post-graft.

**Figure 16:**
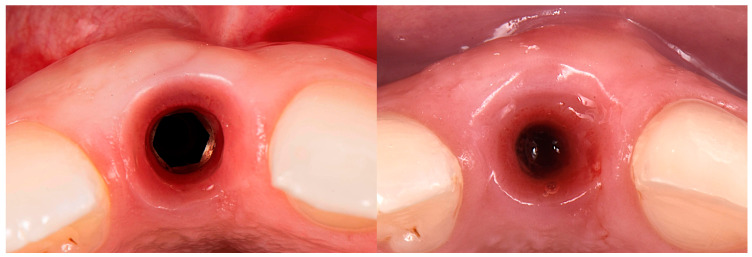
Occlusal view pre- and 6 months post-graft.

**Figure 17:**
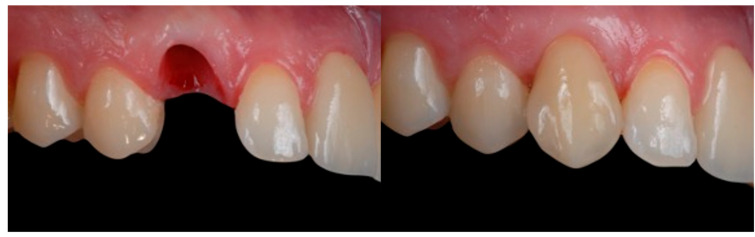
Frontal image pre- and 6 months post-graft.

The results obtained in this present study provide relevant evidence regarding the effectiveness of the trouser-shaped connective tissue graft technique for peri-implant soft tissue augmentation. A statistically significant volume gain was observed at both coronal and buccal levels, with means of 2.74 mm and 2.79 mm, respectively. This gain is also clinically relevant in terms of peri-implant tissue stability and aesthetics (Figure 13, Figure 14, Figure 15, Figure 16 and Figure 17).

When analyzing the results by evaluated zones, a greater increase in thickness was detected in the medial area of the middle region. This is likely because this area corresponds to the center of the graft, where a larger volume of tissue is concentrated. Similarly, in the papillae, the area with the greatest thickening was closest to the implant’s emergence profile, i.e., the center of the graft, while at the extremities, closer to the adjacent teeth, the volume gain was smaller.

Comparing these findings with those observed in the literature, we can see that they partially align with other studies. Eeckhout et al. reported a mean horizontal volume gain of 1.2 ± 0.7 mm in the buccal zone at three years [27]. In another study by Thoma et al., a similar increase in soft tissue thickness was shown at 90 days, with a median of 1.3 mm in both the collagen matrix and autologous graft groups [4]. Hosseini et al. observed stable volumetric improvement in the anterior maxilla, although the augmentation was limited to the buccal zone [28].

It should be noted that many studies employ methods based on linear measurements with a periodontal probe and an analysis of standardized clinical photographs, without three-dimensional reconstruction, so data comparison must be approached with some caution [29,30]. In this study, STL file superimposition was performed, a technique that has been consolidated in the recent literature as the method of choice, offering non-invasive and high-precision measurement, surpassing traditional techniques in reliability [31,32].

Regarding the follow-up time to assess treatment effectiveness, it varies greatly in the literature. The 6 months of observation established in this study can be considered sufficient based on the work of Lin et al., which indicates that the main parameters related to soft tissue stability (such as keratinized tissue width or mucosal recession) stabilize approximately three months after surgery [33]. Nevertheless, other research, including studies by Zuiderveld et al. (2021, 2025), has reported no statistically significant differences in aesthetic outcomes at one-year and five-year assessments [34,35].

Compared to other described techniques, one of the main advantages of the technique presented in this study is the possibility of increasing both buccal and interproximal tissue volume in a single intervention using a single donor site thanks to the graft’s design and manipulation. Furthermore, by employing a tunneling technique instead of incisions in the papillae, physiological tissue contraction is minimized.

Regarding morbidity, no clinical complications were recorded. This can be attributed to both the use of tunneling and the choice of the tuberosity as the donor site, factors that are usually associated with less postoperative discomfort [36,37]. Mancini et al. in 2023 highlight that appropriate indication and timing for soft tissue augmentation contribute to reducing the complication rate [38]. Additionally, factors such as operator experience, implant position, and patient expectations should be considered.

Finally, it is important to acknowledge the limitations of this study, as the low sample size may compromise the robustness of the statistical results and might affect optimal statistical power. The results should be interpreted with caution due to the short follow-up period, absence of a control group, and the inherent limitations of a Likert scale, such as the potential for response biases, the difficulty in capturing nuances in complex opinions, and the challenge in inferring the reasons behind the responses. It is important to consider that methodological differences, study design, follow-up duration, and interindividual variability can influence the comparison of results among different investigations.

## 6. Conclusions

The Trouser Technique with connective tissue grafting is a predictable and minimally invasive alternative to manage volume defects in peri-implant soft tissues, especially in aesthetic areas.

Its design allows for simultaneous vestibular and interproximal thickness augmentation in a single procedure and from a single donor site, which reduces postoperative morbidity and promotes greater patient acceptance. It is particularly valuable in the anterior region, where stability of the emergence profile and soft tissue volume are critical to long-term clinical success and patient satisfaction.

A statistically significant soft tissue volume gain was observed at both the coronal and vestibular levels in all positions tested (*p* < 0.001). Therefore, future studies with larger sample sizes and longer follow-ups are necessary to validate these findings and establish more robust clinical recommendations since this present study has a short follow-up period.

## Figures and Tables

**Table 1:** Mean gain (mm) and its 95% confidence intervals at coronal and vestibular levels (general and by location/zone).

Level	Category	N	Media	IC 95%	*p*-Value
Coronal	Global	90	2.74	2.21–3.26	<0.001
	Mesial papila	30	2.66	2.23–3.10	<0.001
	Medial	30	2.77	2.24–3.33	<0.001
	Distal papila	30	2.76	2.16–3.37	<0.001
	Mesial zone	30	2.63	2.12–3.13	<0.001
	Medial zone	30	2.83	2.33–3.32	<0.001
	Distal zone	30	2.76	2.19–3.33	<0.001
Vestibular	Global	90	2.79	2.24–3.35	<0.001
	Mesial papila	30	2.74	2.29–3.19	<0.001
	Medial	30	2.88	2.36–3.39	<0.001
	Distal papila	30	2.77	2.07–3.46	<0.001
	Mesial zone	30	2.71	2.17–3.25	<0.001
	Medial zone	30	2.87	2.38–3.36	<0.001
	Distal zone	30	2.81	2.19–3.42	<0.001

## Data Availability

The original contributions presented in this study are included in the article/Supplementary Material. Further inquiries can be directed to the corresponding author.

## Supplementary Materials

The following supporting information can be downloaded at: https://www.mdpi.com/article/10.3390/jcm14144974/s1, Video S1: Graft placement; Video S2: Preparation of the graft.
